# Neural Correlates of Emotional Interference in Social Anxiety Disorder

**DOI:** 10.1371/journal.pone.0128608

**Published:** 2015-06-04

**Authors:** Stephanie Boehme, Viktoria Ritter, Susan Tefikow, Ulrich Stangier, Bernhard Strauss, Wolfgang H. R. Miltner, Thomas Straube

**Affiliations:** 1 Laboratory of Psychophysiology and functional Imaging, Department of Psychiatry, Psychosomatics & Psychotherapy, University Hospital Würzburg, Fuechsleinstr. 15, D-97080 Würzburg, Germany; 2 Department of Biological and Clinical Psychology, Friedrich Schiller University Jena, Am Steiger 3 // 1, D-07743 Jena, Germany; 3 Department of Clinical Psychology and Psychotherapy, Goethe-University Frankfurt, Varrentrappstr. 40–42, D-60486 Frankfurt am Main, Germany; 4 Institute of Psychosocial Medicine and Psychotherapy, Jena University Hospital—Friedrich Schiller University, Stoystr. 3, D-07740 Jena, Germany; 5 Institute of Medical Psychology and Systems Neuroscience, University of Muenster, Von-Esmarch-Str. 52, D-48149 Muenster, Germany; University of Maryland, College Park, UNITED STATES

## Abstract

Disorder-relevant but task-unrelated stimuli impair cognitive performance in social anxiety disorder (SAD); however, time course and neural correlates of emotional interference are unknown. The present study investigated time course and neural basis of emotional interference in SAD using event-related functional magnetic resonance imaging (fMRI). Patients with SAD and healthy controls performed an emotional stroop task which allowed examining interference effects on the current and the succeeding trial. Reaction time data showed an emotional interference effect in the current trial, but not the succeeding trial, specifically in SAD. FMRI data showed greater activation in the left amygdala, bilateral insula, medial prefrontal cortex (mPFC), dorsal anterior cingulate cortex (ACC), and left opercular part of the inferior frontal gyrus during emotional interference of the current trial in SAD patients. Furthermore, we found a positive correlation between patients’ interference scores and activation in the mPFC, dorsal ACC and left angular/supramarginal gyrus. Taken together, results indicate a network of brain regions comprising amygdala, insula, mPFC, ACC, and areas strongly involved in language processing during the processing of task-unrelated threat in SAD. However, specifically the activation in mPFC, dorsal ACC, and left angular/supramarginal gyrus is associated with the strength of the interference effect, suggesting a cognitive network model of attentional bias in SAD. This probably comprises exceeded allocation of attentional resources to disorder-related information of the presented stimuli and increased self-referential and semantic processing of threat words in SAD.

## Introduction

Individuals suffering from social anxiety disorder (SAD) showed biased processing of disorder-related information (e.g. [1,2]). In this context, several studies have shown that SAD patients exhibit strong sensitivity towards disorder-related cues, such as socially-threatening words or aversive facial expressions [3,4]. Along these lines, attentional bias, i.e. the capture of attention and interference by threat-related information, has particularly been investigated in SAD patients [3]. This bias has been suggested to play a central role in the disorder’s development and maintenance [5,6].

The emotional stroop task is often used to quantify attentional bias. In this task, the emotional stroop interference is reflected by longer reaction times in ink color naming of disorder-related in comparison to neutral words. Fast and automatic allocation of attention to and prioritized processing of disorder-related words has been suggested to interfere with the cognitive task and thus to cause increased reaction times (e.g. [7,8]). In general, patients with anxiety disorders show increased reaction times to disorder-relates cues in emotional stroop tasks (see [8]). There is evidence for emotional stroop interference in SAD patients, too (e.g. [9–13]; but see also [14–16]). However, during the processing of generally threatening in comparison to neutral words subjects without an anxiety disorder also show an interference effect that seems to be at least partly related to carry-over effects (e.g. [17]) or slow emotional stroop effects, respectively (e.g. [18,19]). Such slow effects are evident in those trials that follow threat-related trials. The relevance of fast and slow emotional interference effects in patients with anxiety disorders is completely unknown.

Furthermore, there is a lack of studies on the neural correlates of emotional interference in SAD. Generally, the amygdala, the insula, the anterior cingulate cortex (ACC), and other frontal brain areas were shown to be activated during processing of threat-related information in patients with SAD (e.g. [20–23]). Subcortical areas, such as the amygdala, have been proposed to mediate automatic, bottom-up processing of emotional, and especially of threatening stimuli [24]. With its interconnections to various cortical areas as well as the brain stem and the hypothalamus, the amygdala plays a central role in alerting responses, the regulation of the autonomic nervous system, and the modulation of the perceptual and emotional processing of threatening and fearful stimuli [25–27]. Furthermore, elevated amygdala reactivity might be a premorbid risk factor regarding stress-related disorders (see [28]). Areas that are involved in the representation of bodily states such as the insula have been shown to be more related to explicit processing of threat and a person’s own bodily states (e.g. [21,29,30]).

Medial areas of the prefrontal cortex, including the ACC have been suggested to be involved in cognitive-emotional interactions (e.g. [31]). These areas were considered to be relevant for higher cognitive appraisal processes, including the experience but also the control and regulation of emotional responses. Previous studies in healthy subjects showed that rostral and dorsal parts of the ACC were activated during emotional interference tasks (e.g. [32–40]). This was suggested to reflect demands in cognitive control and emotion regulation [41]. In patients with anxiety disorders, difficulties in controlling emotional distraction or conflict have been proposed, which might be related to insufficient down-regulation of amygdala hyperactivation [42–44].

Emotional interference in distraction tasks in individuals with anxiety disorders should be mainly caused by the fact that task-unrelated but disorder-relevant information capture attention and processing resources, thus interfering with the main task (e.g. [45]). In the emotional stroop paradigm, the task-unrelated presence of threatening words should be associated with increased emotional, perceptual, and semantic processing of this disorder-related information, and thus with increased responses in amygdala, insula and medial prefrontal areas as well as language areas that are implemented in word-processing. Emotional words have been shown to increase activation in several left hemispheric word-processing areas, including the opercular part of the inferior frontal gyrus (IFG) which includes Broca’s area [46], fusiform gyrus (FG) [47], angular, and supramarginal gyrus, including Wernicke’s area and adjacent brain regions [48].

The present study investigated the neural correlates of emotional interference during an event-related emotional stroop task in patients with SAD and healthy subjects. The current paradigm allowed the investigation of fast and slow interference effects, i.e. of the current trial as well as the potential carry over effect to the succeeding trial. In particular, the present study aimed to answer three main questions: 1) What is the time course of emotional interference in patients with SAD? 2) Which brain areas are involved in the processing of threat during the emotional stroop paradigm in SAD? 3) Which brain areas are specifically associated with the interference effects?

## Materials and Methods

### Subjects

Seventeen patients with SAD and 16 healthy control subjects (HC) participated in the study. Due to exceeding head movements (> 3 mm) in the scanner, one patient had to be excluded from analyses. Therefore, final samples under study consist of 16 SAD and 16 HC subjects. All were right-handed with normal or corrected-to-normal vision. Participants were recruited via public announcement and all provided written informed consent to participate in the study. The study was approved by the ethics committee of the University of Jena. SAD diagnoses were confirmed by Structured Clinical Interview for DSM-IV Axis I and II disorders (SCID I and II; [49,50]). Exclusion criteria were any of the following: 1) a diagnosis of obsessive-compulsive disorder, psychotic disorder or dementia, or current primary or secondary major depression; 2) a history of seizures or head injury with loss of consciousness; 3) a severe uncontrollable medical condition; or 4) the use of any psychotropic medication within the preceding six months. In the SAD sample, comorbidities were agoraphobia with panic disorder (n = 2), affective disorder (major depressive disorder, in full remission; n = 10), and bulimia nervosa (n = 1). Three patients previously suffered from alcohol and/or substance abuse. Additionally, criteria of DSM-IV-TR axis II personality disorders were fulfilled by eight patients (six with avoidant and two with dependent personality disorder). HC subjects were free of any psychopathology. SAD and HC individuals were matched for age, education, and gender (see Table 1). After scanning, participants completed the LSAS [Liebowitz Social Anxiety Scale, German version; [51]) and the BDI (Beck Depression Inventory, German version; [52]) questionnaires. SAD patients scored significantly higher on both LSAS and BDI questionnaires than HC subjects (Table 1).

**Table 1:** Demographic and questionnaire characteristics for patients with social anxiety disorder (SAD) and healthy control subjects (HC) concerning gender, age, education, symptom severity (LSAS), and depression (BDI).

	SAD	HC	t-value/χ^2^-value
sex, No.			.14
female	6	5	
male	10	11	
age, y	29.06 ± 9.84	30.81 ± 8.83	.53
(range)	(19–54)	(19–47)	
education, y	11.25 ± 1.00	11.33 ±. 98	.24
(range)	(10–12)	(10–12)	
LSAS	83.69 ± 19.73	19.38 ± 9.59	11.73*
(range)	(59–118)	(3–35)	
BDI	10.28 ± 5.45	4.50 ± 4.26	3.35*
(range)	(2–18)	(0–16)	

* p < .05; y = years

Mean ± standard deviation; range displayed in parentheses

### Paradigm

Thirty-six disorder-related (e.g. “speech”, “to blush”, “awkward”) and 36 neutral words (e.g. “garage”, “to size”, “movable”; a subset of words that were already used in one of our previous studies; see [21]) matched with respect to the number of syllables and word frequency in German language (COSMAS II; version 3.6.1, Institute for German language, Mannheim, Germany) were used.

Two functional magnetic resonance imaging (fMRI) runs followed an anatomical scan. During the fMRI runs, all words were shown once and in a pseudo random order (no more than three succeeding words of the same category) on an overhead mirror for 1 s with an inter trial interval of 3.5 to 5.5 s in which a white fixation cross on a black background was presented. Words were printed in green, red, blue, or yellow color on a black background. Participants were requested to name the ink color of the words as fast as possible by pressing one of four buttons with either the index or middle finger of the right or left hand. Subjects’ responses were recorded with an optic response box. The order of the two runs, the four ink colors, and the assignment of the response buttons to the four fingers was counterbalanced across individuals. The pseudo randomization of stimulus order was used to secure an equal number of stimulus pairs (neutral words with a preceding neutral word [NN]; neutral words with a preceding social word [SN]; social words with a preceding neutral word [NS]; and social words with a preceding social word [SS]). This special randomization allowed analyses of the effect of the actual trial—without being confounded by the preceding word (NS vs. NN)—and the effect of the preceding trial (SN vs. NN), separately. Only correct trials were included in the analysis. There were no effects of Condition or Group on accuracy (all main effects and interactions: *p* > .05; see Table 2 for descriptive scores).

**Table 2:** Reaction times (in ms) and accuracy (in %) scores of the four stimulus pairs (NN: neutral – neutral; NS: neutral – social; SN: social – neutral; SS: social – social) in patients with social anxiety disorder (SAD) and healthy control (HC) subjects (standard deviation are displayed in parentheses).

	SAD				HC			
	NN	NS	SN	SS	NN	NS	SN	SS
reaction times	862.96	905.44	882.15	874.22	741.59	749.84	760.73	754.11
	(181.03)	(225.86)	(239.13)	(178.34)	(133.95)	(146.35)	(179.72)	(168.77)
accuracy	97.71	95.94	97.88	95.77	98.60	97.54	97.19	98.06
	(3.71)	(3.85)	(3.51)	(4.23)	(1.77)	(3.38)	(3.56)	(2.66)

After scanning, participants rated all words using a nine-point Likert scale (SAM, Self Assessment Manikin; [53]) to assess valence (1 = very pleasant to 9 = very unpleasant, whereas 5 = neutral), arousal (1 = not arousing to 9 = very arousing), and threat (1 = not threatening to 9 = very threatening). Behavioral data were analyzed by repeated measures analysis of variance (ANOVA) and t-tests using the software SPSS (Version 18.0.2; SPSS, INC.). A probability level of *p* < .05 was considered statistically significant.

### FMRI

MRI data were recorded with a 3 Tesla magnetic resonance scanner (“Magnetom TIM TRIO”, Siemens, Medical Solutions, Erlangen, Germany). Scanning consisted of a T1-weighted anatomical scan and two runs using T2*-weighted echo-planar sequence. Each run comprised 135 volumes (TE_echo time_ = 30 ms, flip angle = 90°, matrix = 64 x 64, FOV_field of view_ = 192 mm, TR_repetition time_ = 3 s). Volumes consisted of 40 axial slices (thickness = 3 mm, gap = 0 mm, in plane resolution = 3 x 3 mm, slice order = ascending). The first four volumes of each run were skipped to secure steady-state tissue magnetization.

The software package BrainVoyager QX software (Version 1.10.4; Brain Innovation, Maastricht, The Netherlands) was used for data preprocessing and analyzing. To minimize artifacts due to participants’ head movements during scanning, all volumes of each run were realigned to the first volume. Then a slice time correction was conducted. Further, data was spatially filtered (8 mm full-width half-maximum isotropic Gaussian kernel) and temporally smoothed (high pass filter: 5 cycles per run; low pass filter: 2.8 s; linear trend removal). Finally, the anatomical and functional images were co-registered and normalized to the Talairach space [54].

For statistical analyses, a multiple linear regression modelling the signal time course at each voxel was calculated. The expected BOLD (blood oxygen level-dependent) signal change for each predictor was modeled with a canonical hemodynamic response function. Predictors of interest were the four stimulus pairs NN, NS, SN, and SS. Motion correction parameters were defined as events of no interest. Statistical comparisons were conducted using a mixed effect analysis, which considers inter-subject variance and permits population-level inferences. Voxel-wise statistical maps were generated and the predictor estimates (beta-weights) were computed for each individual. Two ANOVAs (repeated measures)—one for fast stroop effect (NN vs. NS) and one for slow stroop effect (NN vs. SN)—with NN vs. NS/ NN vs. SN as within-subject factors and with group (SAD vs. HC) as between subject factor were conducted. Analyses were performed for specific regions of interest (ROIs). Following the approach recommended by Eickhoff et al. [55], we extracted the amygdala ROI consisting of three bilateral amygdala maximum probability maps (laterobasal, centromedial, and superficial; 9,077 mm^3^ in total) of the anatomy toolbox [56]. ROIs for the bilateral insula (32,822 mm^3^), ACC (60,595 mm^3^), left opercular IFG (9,585 mm^3^), left FG (21,038 mm^3^), left angular gyrus (10,406 mm^3^), and left supramarginal gyrus (11,383 mm^3^) were extracted from the AAL atlas included in WFU PickAtlas software [57–59]. Using MATLAB (Version 7.8; The MathWorks, Inc) all maps were transformed into BV-compatible Talairach coordinates via ICBM2tal [60]. The ROI for mPFC (28,392 mm^3^) was designed by using the BrainVoyager software and creating a cube of 26 mm diameter around x = +/-5; y = 46; z = 18 Talairach coordinates (see [61,62]). Finally, correlation analyses were conducted between brain activation within the ROIs and patients’ reaction time differences.

Statistical parametric maps resulting from voxel-wise analyses were considered statistically significant for clusters that survived cluster-based correction for multiple comparisons (as implemented in BrainVoyager; see [63]; and that was based on a 3D extension of the randomization procedure described by Forman et al. [64]). For this purpose, the voxel-level threshold was set at *p* < .005 (uncorrected). Then, the threshold maps were submitted to a ROI-based correction for multiple comparisons. The cluster threshold criterion was based on the estimate of map’s spatial smoothness [64] and on an iterative procedure (Monte Carlo simulation). The Monte Carlo simulation used 1,000 iterations to estimate the minimum cluster size threshold. A cluster-level false-positive rate of 5% was used.

## Results

### Behavioral data

#### Reaction times

**Fig 1:**
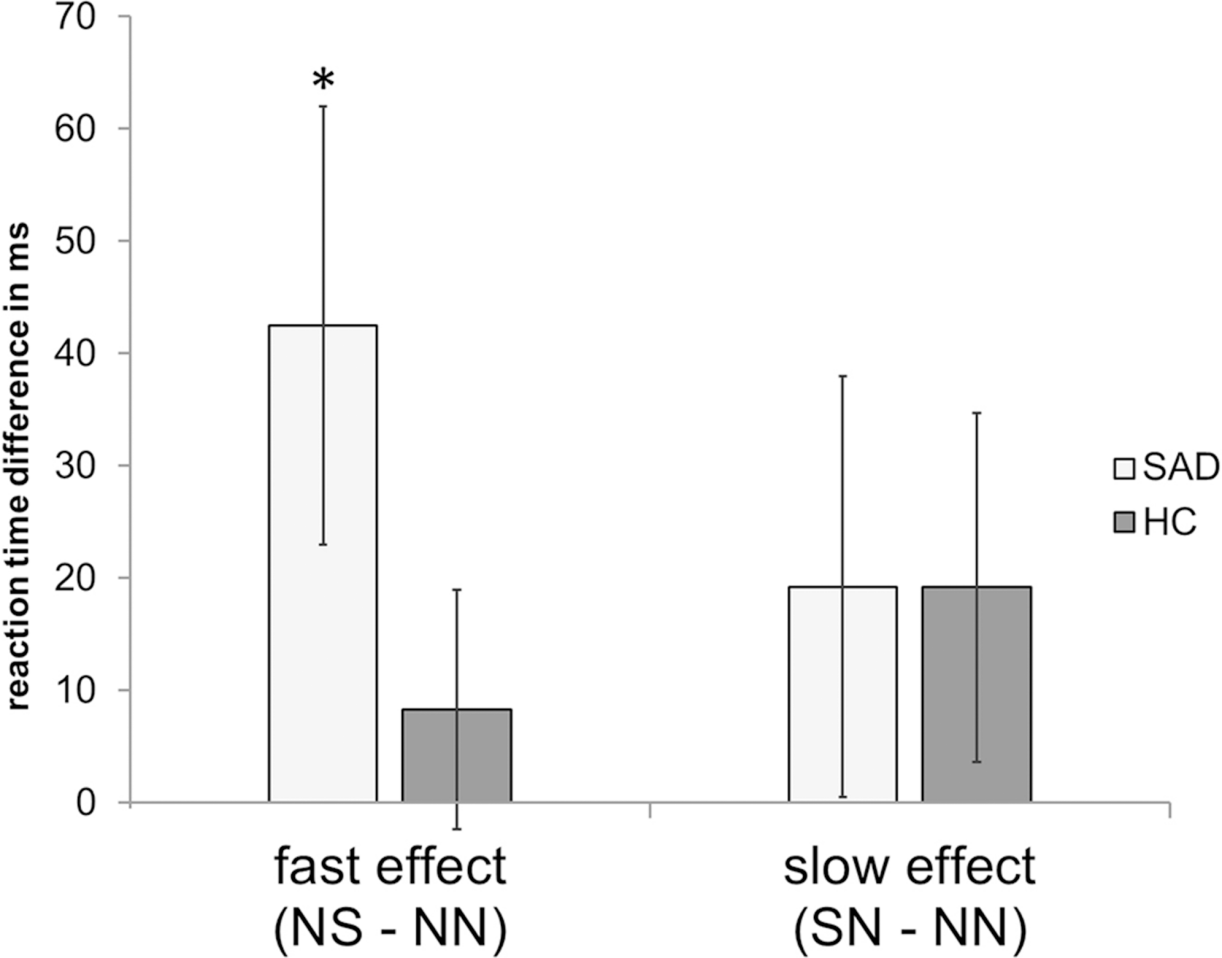
Reaction time differences. Reaction time differences for fast (NS – NN) and slow stroop effect (SN – NN) in patients with social anxiety disorder (SAD) and healthy control subject (HC). * indicates significant differences.

We analyzed reaction time differences in response to social vs. neutral stimuli, which were preceded by trials with neutral stimuli (NS – NN; interference effect of actual threat), and to neutral stimuli with preceding social vs. neutral words (SN – NN; carry-over effect of interference). A significant interference effect is given when the difference between trial types is significantly greater than 0. The only significant interference effect was found for patients with SAD in the actual threat condition (*t*[15] = 2.19, *p* < .05_corr. for multiple comparisons_ stepwise Bonferroni correction; [65]). This effect did not occur in HCs (*t*[15] = .76, *p* > .05_corr_). Carry-over effects were found neither in the SAD nor in the HC group (SAD: *t*[15] = 1.02, *p* > .05_corr_; HC: *t*[15] = 1.23, *p* > .05_corr_). The Group x Condition interaction failed to reach statistical significance (*F*[1,30] = 1.88, *p* = .18). Fig 1 indicates the pattern of results (for descriptive scores see Table 2).

#### Rating data

Analyses of post-scanning rating data showed that both SAD patients and HC subjects rated social relative to neutral words as more unpleasant (*F*[1,31] = 50.69, *p* < .05), more arousing (*F*[1,31] = 73.65, *p* < .05), and more threatening (*F*[1,31] = 65.19, *p* < .05). There was also a significant Word category by Group interaction (unpleasantness: *F*[1,31] = 6.52, *p* < .05; arousal: *F*[1,31] = 8.20, *p* < .05; threatening sentiments: *F*[1,31] = 20.64, *p* < .05). Subsequent t-tests showed that SAD patients rated social words as more unpleasant (*t*[15] = 3.68, *p* < .05), more arousing (*t*[15] = 3.16, *p* < .05), and more threatening (*t*[15] = 4.79, p < .05) than HCs. Ratings of neutral words did not differ between the two groups (unpleasantness: *t*[15] = .19, *p* > .05; arousal: *t*[15] = .09, *p* > .05; threat: *t*[15] = .07, *p* > .05). Table 3 summarizes these results.

**Table 3:** Post scanning rating data of unpleasantness, arousal, and threat to neutral and social words by patients with social anxiety disorder (SAD) and healthy control (HC) subjects (standard deviation are displayed in parentheses).

	SAD		HC	
rating of	neutral	social	neutral	social
*unpleasantness*	3.90	6.73	4.00	5.33
	(1.36)	(1.20)	(1.51)	(0.94)
*arousal*	1.88	5.88	1.85	3.85
	(0.93)	(1.88)	(1.20)	(1.75)
*threat*	1.70	5.84	1.68	2.83
	(0.83)	(1.99)	(1.21)	(1.53)

### FMRI-data

#### Effect of the actual trial

**Fig 2:**
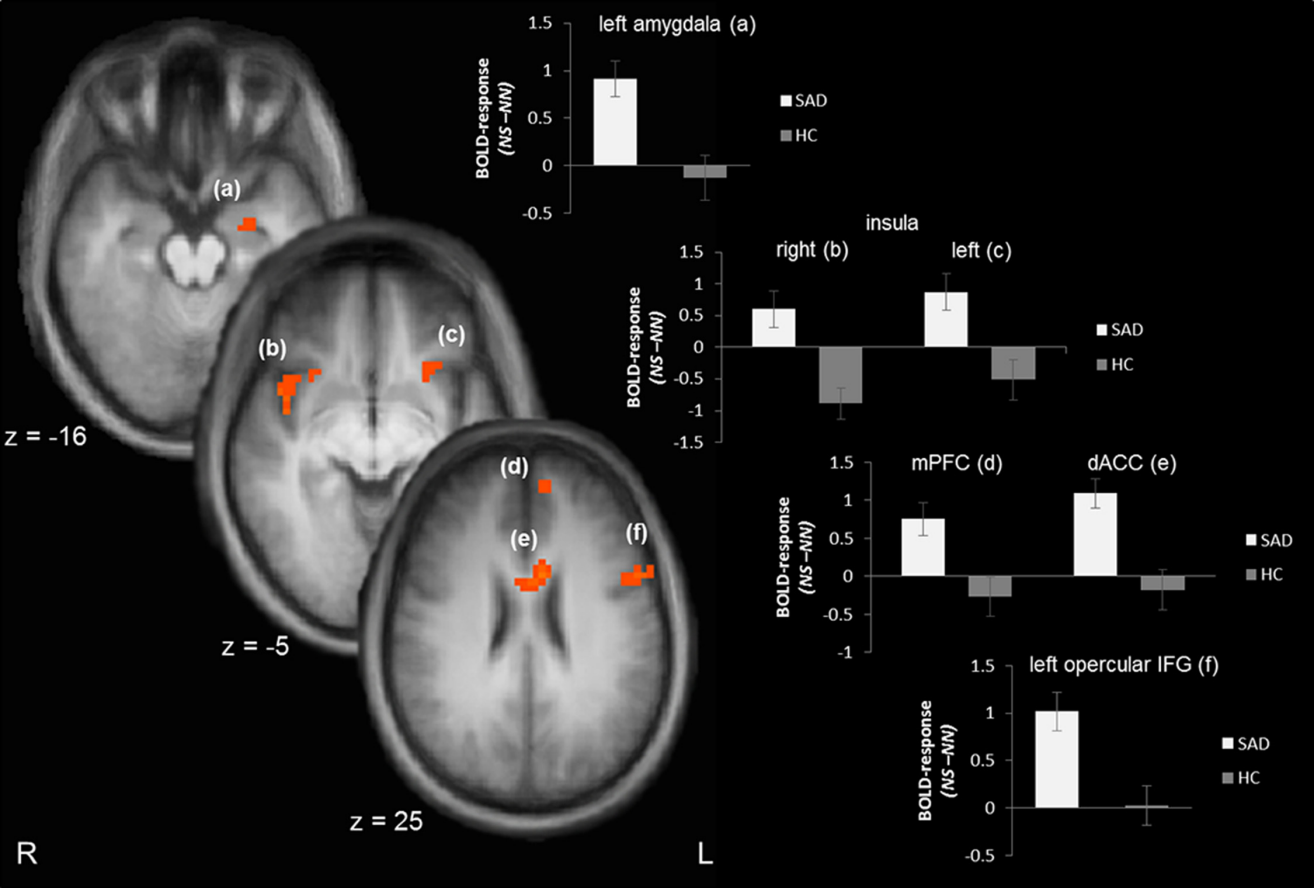
Brain activation differences to the actual trial (NS > NN). Patients with social anxiety disorder (SAD) display an enhanced activation in the left amygdala (a), right (b) and left insula (c), medial prefrontal cortex [mPFC; (d)], dorsal anterior cingulate cortex [ACC; (e)], and left opercular inferior frontal gyrus [IFG; (f)] as compared to healthy control subjects (HC). Statistical parametric maps are overlaid on an averaged T1 scan (radiological convention: left = right). The plots at the right side display contrasts of parameter estimates (mean ± standard error for maximally activated voxel).

Analyses of the fast stroop effect (NS – NN) revealed an increased activation in the left amygdala (peak voxel Talairach coordinates: x = -24; y = -7; z = -16; size = 174 mm^3^; *t*[29] = 3.13; *p* < .05_corr_; probability = 70%), the right and left insula (right: peak voxel Talairach coordinates: x = 43; y = 8; z = -5; size = 814 mm^3^; *t*[29] = 3.71¸ *p* < .05_corr_; left: peak voxel Talairach coordinates: x = -26; y = 15; z = -7; size = 273 mm^3^; *t*[29] = 3.09¸ *p* < .05_corr_), mPFC (peak voxel Talairach coordinates: x = -6; y = 53; z = 25; size = 369 mm^3^; *t*[29] = 3.16¸ *p* < .05_corr_) and the dorsal part of the ACC (peak voxel Talairach coordinates: x = -5; y = 8; z = 28; size = 834 mm^3^; *t*[29] = 4.30¸ *p* < .05_corr_) in SAD patients as compared to HC subjects. Furthermore, a cluster in the left opercular part of the IFG (peak voxel Talairach coordinates: x = -47; y = 12; z = 11; size = 1336 mm^3^; *t*[29] = 3.79¸ *p* < .05_corr_) was significantly stronger activated. There was also a cluster of activated voxels that spread into the left angular gyrus. But the number of activated voxels in the angular gyrus (29 mm^3^) was smaller than the minimal cluster size for activations in the angular gyrus (86 mm^3^) revealed by Monte Carlo simulation. The majority of activated voxels in this cluster was located inferior to angular gyrus. Fig 2 demonstrates significant differences between SAD and HC subjects in response to the actual socially threatening vs. neutral stimuli.

#### Effect of the preceding trial

Similar to behavioral data, analysis of the effects on the preceding trial (SN – NN) revealed no significant activation difference between the two groups.

#### Correlation analyses

**Fig 3:**
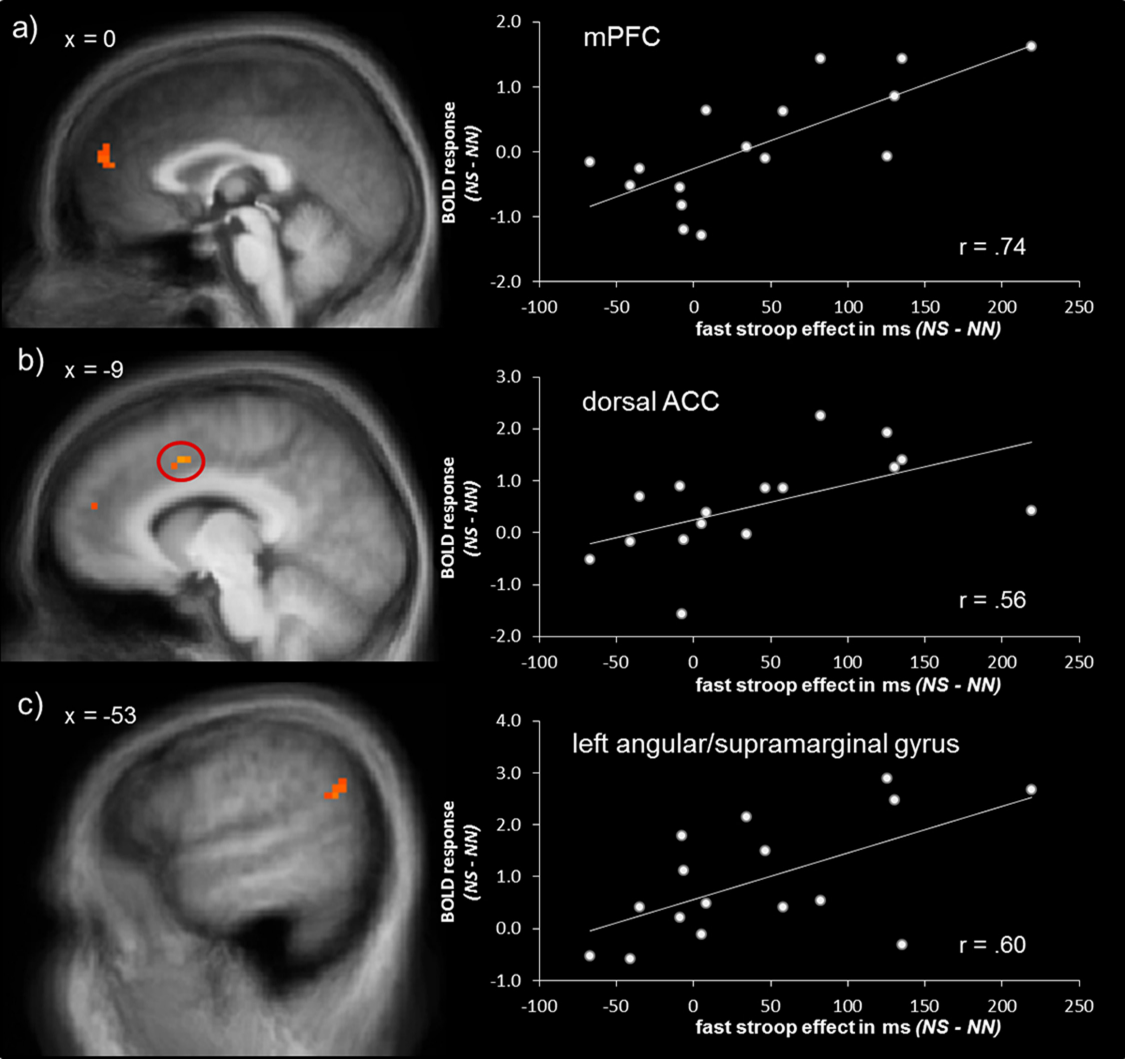
Correlation analyses of brain activation and reaction time differences. The amount of fast stroop effect as measured by reaction time difference (NS – NN) was positively correlated with brain activation in (a) medial prefrontal cortex (mPFC), (b) dorsal ACC, and (c) left angular/supramerginal gyrus in patients with social anxiety disorder. Statistical parametric maps are overlaid on an averaged T1 scan. The scatter plots at the right side display the relationship between contrasts of parameter estimates (NS – NN) and means of reaction time differences (NS – NN).

Correlation analyses of the fast stroop interference effect as measured by reaction time differences of NS – NN with BOLD activation showed a positive association of reaction time differences with activation in mPFC (peak voxel Talairach coordinates: x = 15; y = 44; z = 22; size = 1071 mm^3^; *r* = .74; *p* < .05_corr_; see Fig 3A) and dorsal ACC (peak voxel Talairach coordinates: x = -6; y = 9; z = 41; size = 147 mm^3^; *r* = .56; *p* < .05_corr_; see Fig 3B). There was also a cluster of correlated voxels which spread the border of left angular and supramarginal gyrus (total size = 779 mm^3^) with center of gravity (size and peak voxel) in the left supramarginal gyrus (peak voxel Talairach coordinates: x = -56; y = -54 z = 26; size = 423 mm^3^; *r* = .63; *p* < .05_corr_; see Fig 3C). These correlations did not reflect a correlation with reaction time difference regardless of the interference effect, since there was no significant association between differential reaction times and brain activation in HCs (same peak voxel Talairach coordinates as in SAD patients; mPFC: *r* = -.38, *p* > .05; dorsal ACC: *r* = -.08, *p* > .05; left angular/supramarginal gyrus: *r* = -.15, *p* > .05).

## Discussion

The present study investigated the time course of emotional interference and its neural correlates in SAD patients using a modified emotional stroop task. Behavioral data indicate that SAD patients solely showed emotional stroop interference to current disorder-related words without transmission to the succeeding trial. FMRI analyses revealed stronger activation of several emotion-associated brain regions, such as left amygdala, bilateral insula, mPFC, and dorsal ACC to current social vs. neutral words in SAD vs. HC subjects. Additionally, a brain region that crucially is involved in language processing, the operular IFG containing Broca’s area, was also hyperactivated in SAD patients as compared to healthy subjects. Correlational analyses showed that the strength of the fast interference effect was positively associated with the activation in mPFC, dorsal ACC, and left angular gyrus in SAD patients.

Behavioral results are in accordance with previous studies showing emotional stroop interference in SAD patients (e.g. [9–11,66,67]). Here, we provide evidence that the interference effect in a typical event-related experimental design occurs in the current trial only, and that there is no significant carry over phenomenon or slow effect as has been discussed for emotional stroop tasks [17,18]. The computational model of Wyble and colleagues [68] proposes fast as well as slow stroop interference effects in anxious subjects. However, carry over effects are typically found in designs with very brief inter stimulus intervals. Furthermore, it is conceivable that patients with SAD do not display a slow effect because of avoiding (the emotional meaning of) disorder-related words after initial hypervigilance (e.g. [69,70]).

FMRI data showed that the processing of threat-related words is associated with an increased activation in the left amygdala in individuals with SAD. In SAD patients, an automatic increased amygdala activation to threatening cues, especially to emotionally aversive facial expressions (e.g. [20,70–74]), but also to socially threatening words was found [21]. Furthermore, our finding is in accordance with the assumption of a threat processing system that directs attention automatically to information of potential harm [75,76]. This increased attention towards threatening stimuli has been proposed to lead to the observed extended reaction times in emotional stroop (for an overview see [8]) and other interference tasks. However, we found no evidence for a direct link between amygdala activation and the strength of emotional interference. Instead, for words, regions involved in semantic processing and executive areas seem to be associated with this effect, as discussed below.

Moreover, a cluster in the left and right insula was more activated during the color-naming of disorder-related vs. neutral words in SAD patients. The insular cortex was proposed to be generally involved in anxiety disorders [22,30,77]. Based on theories that proposed insular involvement in the representation of visceral and autonomic responses to emotional stimuli (e.g. [78,79]), increased activation might indicate exaggerated processing of bodily sensations in patients with SAD [6,80], which is in accordance with previous studies that showed insula hyperactivation in SAD [21,22].

There was also a significant activation difference in the opercular part of the IFG including Broca’s area which is an important anterior language area. This is in accordance with previous studies yielding opercular IFG hyperactivation to negative vs. neutral word processing [81,82]. Furthermore, activation of the left angular/supramarginal gyrus was positively correlated with reaction time differences of fast stroop effect in SAD but not in HC subjects. This region was shown to be involved in semantic processing and reading comprehension of words (e.g. [83]), suggesting enhanced semantic analysis of threat-related words [48] during enhanced emotional interference. Our results indicate that the semantic meaning of disorder-related words, although not attended to, seems to be processed in more detail by the SAD patients.

Prefrontal areas were also involved during the processing of threat words. SAD patients showed a hyperactivation of the dorsal ACC. Additionally, dorsal ACC activation was positively correlated with reaction time differences of emotional stroop interference in SAD patients. This region was suggested to be important in tasks requiring selective attention [31,84]. Dorsal ACC activation seems to reflect cognitive and executive demand [37,85,86], but also general arousal regulation [87]. As in previous studies with healthy participants [37–39], interference was associated with activation in dorsal ACC, which may indicate heightened conflict monitoring (see [85]) and task effort [88] due to competing emotional distractors.

There was also hyperactivation of the mPFC to disorder-related vs. neutral words in SAD as compared to HC subjects. Furthermore, mPFC activation was positively correlated with emotional stroop interference in SAD patients. The pregenual area of the mPFC is associated with self-referential [61,89–91] and higher evaluation processes, and also with attentional and emotional control functions [20,32,38,92,93]. The detected association of mPFC activation and reaction time differences suggest that the extent of evaluative and self-referential processes in response to threatening words in SAD patients predicts the strength of emotional interference on the color detection task. Thus, these outcomes do not support the hypothesis of a negative association between interference scores and brain responses in prefrontal cortex as proposed by theories suggesting decreased prefrontal control as a main source of emotional distractibility in anxiety (e.g. [32,93]). Results thus might depend strongly on the applied paradigm. At least for a word-based emotional stroop paradigm as used in the present study, interference effects might be associated with generally increased processing demand in several cortical as well as subcortical structures.

There are some limitations of our study that we would like to note. The study aimed the investigation of the discrete effect of fast as well as slow emotional stroop effects. Nevertheless, with the present study we are not able to answer the question whether slow emotional stroop effects were not observed due to the experimental paradigm that was used. Future studies should investigate the effect of different durations of inter stimulus intervals. Moreover, sample size (sixteen patients) is a further limitation of the present study. Future studies should investigate neural correlates of emotional interference in SAD and generally in anxiety disorders with increased sample size.

## Conclusions

To conclude, the present findings show that threat-related words induce emotional interference in patients with SAD during the current but not the subsequent trial in an emotional stroop task. The processing of disorder-related words was associated with activation in amygdala, insula, prefrontal areas, and brain regions that are essential in language processing. Specifically, activation in mPFC, dorsal ACC, and left angular/supramarginal gyrus was correlated with the strength of emotional interference. Thus, our findings suggest a cognitive network model involving different brain regions that cause increased interference by disorder-related information in SAD. This fast interference seems to be caused by exceeded self-referential as well as semantic processing of social linguistic threat, although semantic information of the stimuli was not task-relevant. Attentional bias has been shown to be associated with the maintenance of anxiety disorders and targeting attentional bias in psychotherapeutic interventions seems to be a promising therapeutic tool in the treatment of anxiety disorders (e.g. [94]). Future studies should investigate whether fast interference effects in emotional stroop like designs are predictive for therapeutic success, altered by psychotherapy and what kind of attentional bias training is most effective as a therapeutic tool for the treatment of SAD.

## References

[1] Spokas ME, Rodebaugh TL, Heimberg RG (2004) Cognitive biases in social phobia. Psychiatry 6: 204–210.

[2] Hirsch CR, Clark DM (2004) Information-processing bias in social phobia. Clinical Psychology Review 24: 799–825. 15501557 10.1016/j.cpr.2004.07.005

[3] Heinrichs N, Hofmann SG (2001) Information processing in social phobia: a critical review. Clinical Psychology Review 21: 751–770. 11434229 10.1016/s0272-7358(00)00067-2

[4] Hirsch CR, Clark DM, Mathews A (2006) Imagery and Interpretations in Social Phobia: Support for the Combined Cognitive Biases Hypothesis. Behavior Therapy 37: 223–236. 16942974 10.1016/j.beth.2006.02.001

[5] Beck AT, Emery G, Greenberg RL (1985) Anxiety Disorders and Phobias: A Cognitive Perspective New York: Basic Books.

[6] Clark DM, Wells A (1995) A cognitive model of social phobia In: Heimberg RG, Liebowitz MR, Hope DA, Schneier FR, editors. Social Phobia: Diagnosis, Assessment, and Treatment. New York: Guilford Press pp. 69–93.

[7] Phaf RH, Kan KJ (2007) The automaticity of emotional Stroop: a meta-analysis. J Behav Ther Exp Psychiatry 38: 184–199. 17112461 10.1016/j.jbtep.2006.10.008

[8] Williams JM, Mathews A, MacLeod C (1996) The emotional Stroop task and psychopathology. Psychological bulletin 120: 3–24. 8711015 10.1037/0033-2909.120.1.3

[9] Gerlach AL, Schiller A, Wild C, Rist F (2006) Effects of alcohol on the processing of social threat-related stimuli in socially phobic women. British Journal of Clinical Psychology 45: 279–295. 17147096 10.1348/014466505x49862

[10] Amir N, Freshman M, Foa E (2002) Enhanced Stroop interference for threat in social phobia. Journal of Anxiety Disorders 16: 1–9. 12171210 10.1016/s0887-6185(01)00084-6

[11] Hope DA, Rapee RM, Heimberg RG, Dombeck MJ (1990) Representations of the self in social phobia: Vulnerability to social threat. Cognitive Therapy and Research 14: 177–189.

[12] Mattia JI, Heimberg RG, Hope DA (1993) The revised Stroop color-naming task in social phobics. Behav Res Ther 31: 305–313. 8476405 10.1016/0005-7967(93)90029-t

[13] Becker ES, Rinck M, Margraf J, Roth WT (2001) The emotional Stroop effect in anxiety disorders: General emotionality or disorder specificity? Journal of Anxiety Disorders 15: 147–159. 11442135 10.1016/s0887-6185(01)00055-x

[14] Andersson G, Westöö J, Johansson L, Carlbring P (2006) Cognitive Bias Via the Internet: A Comparison of Web‐Based and Standard Emotional Stroop Tasks in Social Phobia. Cognitive Behaviour Therapy 35: 55–62. 16500777 10.1080/16506070500372469

[15] Amir N, McNally RJ, Riemann BC, Burns J, Lorenz M, Mullen JT (1996) Suppression of the emotional Stroop effect by increased anxiety in patients with social phobia. Behav Res Ther 34: 945–948. 8990547 10.1016/s0005-7967(96)00054-x

[16] Kindt M, Bogels S, Morren M (2003) Processing bias in children with separation anxiety disorder, social phobia and generalised anxiety disorder. Behaviour Change 20: 143–150.

[17] Waters AJ, Sayette MA, Franken IH, Schwartz JE (2005) Generalizability of carry-over effects in the emotional Stroop task. Behav Res Ther 43: 715–732. 15890165 10.1016/j.brat.2004.06.003

[18] McKenna FP, Sharma D (2004) Reversing the emotional Stroop effect reveals that it is not what it seems: the role of fast and slow components. J Exp Psychol Learn Mem Cogn 30: 382–392. 14979812 10.1037/0278-7393.30.2.382

[19] Frings C, Englert J, Wentura D, Bermeitinger C (2010) Decomposing the emotional Stroop effect. Quarterly Journal of Experimental Psychology 63: 42–49. 10.1080/17470210903156594 19691003

[20] Straube T, Kolassa I T, Glauer M, Mentzel H, Miltner W (2004) Effect of task conditions on brain responses to threatening faces in social phobics: an event-related functional magnetic resonance imaging study. Biol Psychiatry 56: 921–930. 15601601 10.1016/j.biopsych.2004.09.024

[21] Schmidt S, Mohr A, Miltner WHR, Straube T (2010) Task-dependent neural correlates of the processing of verbal threat-related stimuli in social phobia. Biological Psychology 84: 304–312. 10.1016/j.biopsycho.2010.03.005 20227458

[22] Etkin A, Wager TD (2007) Functional neuroimaging of anxiety: A meta-analysis of emotional processing in PTSD, social anxiety disorder, and specific phobia. The American Journal of Psychiatry 164: 1476–1488. 17898336 10.1176/appi.ajp.2007.07030504 PMC3318959

[23] Freitas-Ferrari MC, Hallak JCA, Trzesniak C, Filho AS, Machado-de-Sousa JP, Chagas MHN, et al (2010) Neuroimaging in social anxiety disorder: A systematic review of the literature. Progress in Neuro-Psychopharmacology & Biological Psychiatry 34: 565–580. 20206659 10.1016/j.pnpbp.2010.02.028

[24] Öhman A (2005) The role of the amygdala in human fear: Automatic detection of threat. Psychoneuroendocrinology 30: 953–958. 15963650 10.1016/j.psyneuen.2005.03.019

[25] Lipka J, Miltner W, Straube T (2011) Vigilance for threat interacts with amygdala responses to subliminal threat cues in specific phobia. Biological Psychiatry 2011 70: 472–478. 10.1016/j.biopsych.2011.04.005 21601831

[26] LeDoux J (2000) Emotion circuits in the brain. Annu Rev Neurosci 23: 155–184. 10845062 10.1146/annurev.neuro.23.1.155

[27] Tamietto M, de Gelder B (2010) Neural bases of the non-conscious perception of emotional signals. Nat Rev Neurosci 11: 697–709. 10.1038/nrn2889 20811475

[28] Alexander N, Klucken T, Koppe G, Osinsky R, Walter B, Vaitl D, et al (2012) Interaction of the Serotonin Transporter-Linked Polymorphic Region and Environmental Adversity: Increased Amygdala-Hypothalamus Connectivity as a Potential Mechanism Linking Neural and Endocrine Hyperreactivity. Biological Psychiatry 72: 49–56. 10.1016/j.biopsych.2012.01.030 22418015

[29] Straube T, Miltner WHR (2011) Attention to aversive emotion and specific activation of the right insula and right somatosensory cortex. NeuroImage 54: 2534–2538. 10.1016/j.neuroimage.2010.10.010 20946962

[30] Paulus MP, Stein MB (2006) An insular view of anxiety. Biol Psychiatry 60: 383–387. 16780813 10.1016/j.biopsych.2006.03.042

[31] Pessoa L (2008) On the relationship between emotion and cognition. Nat Rev Neurosci 9: 148–158. 10.1038/nrn2317 18209732

[32] Bishop S, Duncan J, Brett M, Lawrence AD (2004) Prefrontal cortical function and anxiety: controlling attention to threat-related stimuli. Nat Neurosci 7: 184–188. 14703573 10.1038/nn1173

[33] Etkin A, Egner T, Peraza DM, Kandel ER, Hirsch J (2006) Resolving emotional conflict: a role for the rostral anterior cingulate cortex in modulating activity in the amygdala. Neuron 51: 871–882. 16982430 10.1016/j.neuron.2006.07.029

[34] Whalen PJ, Bush G, McNally RJ, Wilhelm S, McInerney SC, Jenike MA, et al (1998) The emotional counting stroop paradigm: a functional magnetic resonance imaging probe of the anterior cingulate affective division. Biological Psychiatry 44: 1219–1228. 9861465 10.1016/s0006-3223(98)00251-0

[35] Mohanty A, Engels AS, Herrington JD, Heller W, Ringo Ho M-H, Banich MT, et al (2007) Differential engagement of anterior cingulate cortex subdivisions for cognitive and emotional function. Psychophysiology 44: 343–351. 17433093 10.1111/j.1469-8986.2007.00515.x

[36] Shin LM, Whalen PJ, Pitman RK, Bush G, Macklin ML, Lasko NB, et al (2001) An fMRI study of anterior cingulate function in posttraumatic stress disorder. Biological Psychiatry 50: 932–942. 11750889 10.1016/s0006-3223(01)01215-x

[37] Haas B, Omura K, Constable RT, Canli T (2006) Interference produced by emotional conflict associated with anterior cingulate activation. Cognitive, Affective, & Behavioral Neuroscience 6: 152–156. 10.3758/cabn.6.2.152 17007235

[38] Blair KS, Smith BW, Mitchell DGV, Morton J, Vythilingam M, Pessoa L, et al (2007) Modulation of emotion by cognition and cognition by emotion. NeuroImage 35: 430–440. 17239620 10.1016/j.neuroimage.2006.11.048 PMC1862681

[39] Mitchell DGV, Nakic M, Fridberg D, Kamel N, Pine DS, Blair RJR (2007) The impact of processing load on emotion. NeuroImage 34: 1299–1309. 17161627 10.1016/j.neuroimage.2006.10.012 PMC1909754

[40] Davis KD, Taylor KS, Hutchison WD, Dostrovsky JO, McAndrews MP, Richter EO, et al (2005) Human Anterior Cingulate Cortex Neurons Encode Cognitive and Emotional Demands. The Journal of Neuroscience 25: 8402–8406. 16162922 10.1523/JNEUROSCI.2315-05.2005 PMC6725669

[41] Ray RD, Zald DH (2012) Anatomical insights into the interaction of emotion and cognition in the prefrontal cortex. Neuroscience & Biobehavioral Reviews 36: 479–501. 21889953 10.1016/j.neubiorev.2011.08.005 PMC3244208

[42] Chechko N, Wehrle R, Erhardt A, Holsboer F, Czisch M, Sämann PG (2009) Unstable Prefrontal Response to Emotional Conflict and Activation of Lower Limbic Structures and Brainstem in Remitted Panic Disorder. PLoS ONE 4: e5537 10.1371/journal.pone.0005537 19462002 PMC2680057

[43] Hariri AR, Mattay VS, Tessitore A, Fera F, Weinberger DR (2003) Neocortical modulation of the amygdala response to fearful stimuli. Biol Psychiatry 53: 494–501. 12644354 10.1016/s0006-3223(02)01786-9

[44] Etkin A, Prater KE, Hoeft F, Menon V, Schatzberg AF (2010) Failure of anterior cingulate activation and connectivity with the amygdala during implicit regulation of emotional processing in generalized anxiety disorder. The American journal of psychiatry 167: 545–554. 10.1176/appi.ajp.2009.09070931 20123913 PMC4367202

[45] Eysenck MW, Derakshan N, Santos R, Calvo MG (2007) Anxiety and cognitive performance: Attentional control theory. Emotion 7: 336–353. 17516812 10.1037/1528-3542.7.2.336

[46] Kuchinke L, Jacobs AM, Grubich C, Võ MLH, Conrad M, Herrmann M (2005) Incidental effects of emotional valence in single word processing: An fMRI study. NeuroImage 28: 1022–1032. 16084739 10.1016/j.neuroimage.2005.06.050

[47] Dehaene S, Cohen L (2011) The unique role of the visual word form area in reading. Trends in Cognitive Sciences 15: 254–262. 10.1016/j.tics.2011.04.003 21592844

[48] Hsu DT, Mickey BJ, Langenecker SA, Heitzeg MM, Love TM, Wang H, et al (2012) Variation in the Corticotropin-Releasing Hormone Receptor 1 (CRHR1) Gene Influences fMRI Signal Responses during Emotional Stimulus Processing. The Journal of Neuroscience 32: 3253–3260. 10.1523/JNEUROSCI.5533-11.2012 22378896 PMC3297975

[49] Fydrich T, Renneberg B, Schmitz B, Wittchen HU (1997) Strukturiertes Klinisches Interview für DSM-IV, Achse II (Persönlichkeitsstörungen) Göttingen: Hogrefe.

[50] Wittchen H-U, Wunderlich U, Gruschwitz S, Zaudig M (1997) Strukturiertes Klinisches Interview für DSM-IV, Achse-I (SKID-I) Göttingen: Hogrefe.

[51] Stangier U, Heidenreich T (2005) Liebowitz Social Anxiety Scale In: Scalarum CIP, editor. Internationale Skalen für Psychiatrie (Internatioal Scales for Psychiatry). Weinheim: Beltz pp. 299–306.

[52] Hautzinger M, Bailer M, Worall H, Keller F (1995) Beck-Depressions-Inventar (BDI) Testhandbuch der deutschen Ausgabe. Bern: Huber.

[53] Bradley MM, Lang PJ (1994) Measuring emotion: the Self-Assessment Manikin and the Semantic Differential. J Behav Ther Exp Psychiatry 25: 49–59. 7962581 10.1016/0005-7916(94)90063-9

[54] Talairach J, Tournoux P (1988) Co-Planar Stereotaxic Atlas of the Human Brain 3-Dimensional Proportional System: An Approach to Cerebral Imaging. Stutgart: Thieme

[55] Eickhoff SB, Heim S, Zilles K, Amunts K (2006) Testing anatomically specified hypotheses in functional imaging using cytoarchitectonic maps. NeuroImage 32: 570–582. 16781166 10.1016/j.neuroimage.2006.04.204

[56] Eickhoff SB, Stephan KE, Mohlberg H, Grefkes C, Fink GR, Amunts K, et al (2005) A new SPM toolbox for combining probabilistic cytoarchitectonic maps and functional imaging data. NeuroImage 25: 1325–1335. 15850749 10.1016/j.neuroimage.2004.12.034

[57] Maldjian JA, Laurienti PJ, Burdette JH (2004) Precentral gyrus discrepancy in electronic versions of the Talairach atlas. NeuroImage 21: 450–455. 14741682 10.1016/j.neuroimage.2003.09.032

[58] Maldjian JA, Laurienti PJ, Kraft RA, Burdette JH (2003) An automated method for neuroanatomic and cytoarchitectonic atlas-based interrogation of fMRI data sets. NeuroImage 19: 1233–1239. 12880848 10.1016/s1053-8119(03)00169-1

[59] Tzourio-Mazoyer N, Landeau B, Papathanassiou D, Crivello F, Etard O, Delcroix N, et al (2002) Automated Anatomical Labeling of Activations in SPM Using a Macroscopic Anatomical Parcellation of the MNI MRI Single-Subject Brain. NeuroImage 15: 273–289. 11771995 10.1006/nimg.2001.0978

[60] Lancaster JL, Tordesillas-Gutiérrez D, Martinez M, Salinas F, Evans A, Zilles K, et al (2007) Bias between MNI and Talairach coordinates analyzed using the ICBM-152 brain template. Human Brain Mapping 28: 1194–1205. 17266101 10.1002/hbm.20345 PMC6871323

[61] Amodio DM, Frith CD (2006) Meeting of minds: the medial frontal cortex and social cognition. Nat Rev Neurosci 7: 268–277. 16552413 10.1038/nrn1884

[62] Steele JD, Lawrie SM (2004) Segregation of cognitive and emotional function in the prefrontal cortex: a stereotactic meta-analysis. NeuroImage 21: 868–875. 15006653 10.1016/j.neuroimage.2003.09.066

[63] Goebel R, Esposito F, Formisano E (2006) Analysis of functional image analysis contest (FIAC) data with brainvoyager QX: From single-subject to cortically aligned group general linear model analysis and self-organizing group independent component analysis. Hum Brain Mapp 27: 392–401. 16596654 10.1002/hbm.20249 PMC6871277

[64] Forman SD, Cohen JD, Fitzgerald M, Eddy WF, Mintun MA, Noll DC (1995) Improved assessment of significant activation in functional magnetic resonance imaging (fMRI): use of a cluster-size threshold. Magn Reson Med 33: 636–647. 7596267 10.1002/mrm.1910330508

[65] Benjamini Y, Hochberg Y (1995) Controlling the false discovery rate: a practical and powerful approach to multiple testing London, ROYAUME-UNI: Royal Statistical Society.

[66] Lundh L-G, Öst L-G (1996) Stroop interference, self-focus and perfectionism in social phobics. Personality and Individual Differences 20: 725–731.

[67] McNeil DW, Ries BJ, Taylor LJ, Boone ML, Carter LE, Turk CL, et al (1995) Comparison of social phobia subtypes using Stroop tests. Journal of Anxiety Disorders 9: 47–57. 10.1016/s0887-6185(99)00004-3 10372342

[68] Wyble B, Sharma D, Bowman H (2008) Strategic regulation of cognitive control by emotional salience: A neural network model. Cognition & Emotion 22: 1019–1051.

[69] Vassilopoulos SP (2005) Social Anxiety and the Vigilance-Avoidance Pattern of Attentional Processing. Behavioural and Cognitive Psychotherapy 33: 13–24.

[70] Schulz C, Mothes-Lasch M, Straube T (2013) Automatic neural processing of disorder-related stimuli in Social Anxiety Disorder (SAD): Faces and more. Frontiers in Psychology 4. 10.3389/fpsyg.2013.00282 PMC3662886 23745116

[71] Campbell DW, Sareen J, Paulus MP, Goldin PR, Stein MB, Reiss JP (2007) Time-varying amygdala response to emotional faces in generalized social phobia. Biol Psychiatry 62: 455–463. 17188251 10.1016/j.biopsych.2006.09.017

[72] Cooney RE, Atlas LY, Joormann J, Eugene F, Gotlib IH (2006) Amygdala activation in the processing of neutral faces in social anxiety disorder: is neutral really neutral? Psychiatry Res 148: 55–59. 17030117 10.1016/j.pscychresns.2006.05.003

[73] Phan KL, Fitzgerald DA, Nathan PJ, Tancer ME (2006) Association between Amygdala Hyperactivity to Harsh Faces and Severity of Social Anxiety in Generalized Social Phobia. Biological Psychiatry 59: 424–429. 16256956 10.1016/j.biopsych.2005.08.012

[74] Yoon KL, Fitzgerald DA, Angstadt M, McCarron RA, Phan KL (2007) Amygdala reactivity to emotional faces at high and low intensity in generalized social phobia: a 4-Tesla functional MRI study. Psychiatry Res 154: 93–98. 17097275 10.1016/j.pscychresns.2006.05.004

[75] Öhman A, Soares J (1993) On the automatic nature of phobic fear: conditioned electrodermal responses to masked fear-relevant stimuli. J Abnorm Psychol 102: 121–132. 8436688 10.1037//0021-843x.102.1.121

[76] LeDoux J (2003) The emotional brain, fear, and the amygdala. Cellular and Molecular Neurobiology 23: 727–738. 14514027 10.1023/A:1025048802629 PMC11530156

[77] Rauch SL, Savage CR, Alpert NM, Fischman AJ, Jenike MA (1997) The functional neuroanatomy of anxiety: a study of three disorders using positron emission tomography and symptom provocation. Biol Psychiatry 42: 446–452. 9285080 10.1016/S0006-3223(97)00145-5

[78] Critchley HD, Wiens S, Rotshtein P, Ohman A, Dolan RJ (2004) Neural systems supporting interoceptive awareness. Nat Neurosci 7: 189–195. 14730305 10.1038/nn1176

[79] Damasio AR, Grabowski TJ, Bechara A, Damasio H, Ponto LL, Parvizi J, et al (2000) Subcortical and cortical brain activity during the feeling of self-generated emotions. Nat Neurosci 3: 1049–1056. 11017179 10.1038/79871

[80] Clark DM, McManus F (2002) Information processing in social phobia. Biological Psychiatry 51: 92–100. 11801234 10.1016/s0006-3223(01)01296-3

[81] Maddock RJ, Garrett AS, Buonocore MH (2003) Posterior cingulate cortex activation by emotional words: fMRI evidence from a valence decision task. Human Brain Mapping 18: 30–41. 12454910 10.1002/hbm.10075 PMC6871991

[82] Straube T, Mentzel HJ, Glauer M, Miltner WHR (2004) Brain activation to phobia-related words in phobic subjects. Neuroscience Letters 372: 204–208. 15542241 10.1016/j.neulet.2004.09.050

[83] Seghier ML, Price CJ (2013) Dissociating frontal regions that co-lateralize with different ventral occipitotemporal regions during word processing. Brain and Language 126: 133–140. 10.1016/j.bandl.2013.04.003 23728081 PMC3730055

[84] Weissman DH, Gopalakrishnan A, Hazlett CJ, Woldorff MG (2005) Dorsal Anterior Cingulate Cortex Resolves Conflict from Distracting Stimuli by Boosting Attention toward Relevant Events. Cerebral Cortex 15: 229–237. 15238434 10.1093/cercor/bhh125

[85] Botvinick MM, Braver TS, Barch DM, Carter CS, Cohen JD (2001) Conflict monitoring and cognitive control. Psychological Review 108: 624–652. 11488380 10.1037/0033-295x.108.3.624

[86] Botvinick MM, Cohen JD, Carter CS (2004) Conflict monitoring and anterior cingulate cortex: an update. Trends in Cognitive Sciences 8: 539–546. 15556023 10.1016/j.tics.2004.10.003

[87] Critchley HD, Mathias CJ, Dolan RJ (2001) Neural activity in the human brain relating to uncertainty and arousal during anticipation. Neuron 29: 537–545. 11239442 10.1016/s0896-6273(01)00225-2

[88] Mulert C, Menzinger E, Leicht G, Pogarell O, Hegerl U (2005) Evidence for a close relationship between conscious effort and anterior cingulate cortex activity. International Journal of Psychophysiology 56: 65–80. 15725491 10.1016/j.ijpsycho.2004.10.002

[89] Johnson SC, Baxter LC, Wilder LS, Pipe JG, Heiserman JE, Prigatano GP (2002) Neural correlates of self-reflection. Brain 125: 1808–1814. 12135971 10.1093/brain/awf181

[90] Mitchell JP, Banaji MR, Macrae CN (2005) The Link between Social Cognition and Self-referential Thought in the Medial Prefrontal Cortex. Journal of Cognitive Neuroscience 17: 1306–1315. 16197685 10.1162/0898929055002418

[91] Northoff G, Heinzel A, de Greck M, Bermpohl F, Dobrowolny H, Panksepp J (2006) Self-referential processing in our brain—A meta-analysis of imaging studies on the self. NeuroImage 31: 440–457. 16466680 10.1016/j.neuroimage.2005.12.002

[92] Mechias M-L, Etkin A, Kalisch R (2010) A meta-analysis of instructed fear studies: Implications for conscious appraisal of threat. NeuroImage 49: 1760–1768. 10.1016/j.neuroimage.2009.09.040 19786103

[93] Etkin A, Egner T, Kalisch R (2011) Emotional processing in anterior cingulate and medial prefrontal cortex. Trends in Cognitive Sciences 15: 85–93. 10.1016/j.tics.2010.11.004 21167765 PMC3035157

[94] Bar-Haim Y (2010) Research Review: attention bias modification (ABM): a novel treatment for anxiety disorders. Journal of Child Psychology and Psychiatry 51: 859–870. 10.1111/j.1469-7610.2010.02251.x 20456540

